# Measuring electroretinograms in tree shrews with a portable electrophysiology device

**DOI:** 10.1007/s10633-026-10095-w

**Published:** 2026-03-30

**Authors:** Rohit Dhakal, Nilda C. Sanchez, Timothy J. Gawne, Safal Khanal

**Affiliations:** Department of Optometry and Vision Science, University of Alabama at Birmingham (UAB), 1716 University Blvd., Birmingham, AL 35233 USA

**Keywords:** Electroretinogram, Tree shrews, Emmetropization, Myopia, Animal models

## Abstract

**Purpose:**

The RETeval portable flash electrophysiology device is extensively used for assessing retinal function in humans. However, its utility in measuring electroretinograms in preclinical studies has been less explored. Tree shrews (small diurnal mammals closely related to primates) are a well-established animal model of emmetropization known to exhibit robust responses to visual cues and mimic human juvenile-onset myopia. This study aimed to evaluate the intra- and inter-session repeatability of full-field photopic electroretinograms (ERGs) recorded using the RETeval system in tree shrews.

**Methods:**

At 24 ± 1 days of visual experience (DVE), twelve juvenile tree shrews housed in broadband colony light (100–300 lx) underwent electrophysiology testing of the normally developing eye using the RETeval system (LKC Technologies, MD, USA). Measurements were repeated twice in each session, as well as at 30 and 35 DVE. The amplitudes and peak times of the flash, flicker, photopic negative response, S-cone, and On–Off ERGs were analyzed. The intra- and inter-session repeatability was assessed using the coefficient of variation (CoV), intra-class correlation coefficient (ICC), mean bias (%), paired t-test, and Bland–Altman analysis.

**Results:**

Tree shrew ERGs exhibited well-defined and robust waveforms comparable to those of human ERGs. Flash and flicker ERGs showed excellent intra-session repeatability for both amplitudes and peak times (CoV < 5%, mean bias ≤ 5%, and ICC ≥ 0.98). While the ERGs were also repeatable across different sessions, the repeatability metrics were comparatively better for peak times (CoV: < 10%, mean bias: < 15%) than for amplitudes (CoV: 13.36–29.35%, mean bias: 18.89–41.51%) with ICC values between 0.4 and 0.86 for inter-session ERGs, indicating modest repeatability.

**Conclusions:**

The RETeval measurements of full-field photopic ERGs in tree shrews were robust and reliable, with waveforms morphologically similar to human ERGs, demonstrating the utility of the RETeval system in measuring retinal function in this species. The photopic ERGs were repeatable across sessions on different days, indicating the feasibility of using this device in longitudinal studies of retinal function in tree shrews, for example, in studying the neural basis of emmetropization.

**Supplementary Information:**

The online version contains supplementary material available at https://doi.org/10.1007/s10633-026-10095-w.

## Introduction

Retinal function assessment is an integral component of understanding the health and disorders of the central nervous system, including the retina [1]. A widely used non-invasive method for evaluating retinal function is electroretinography, which provides objective measurements of the neural activity that arises from different retinal cell populations [2]. In vision science research, electroretinograms (ERGs) are routinely used to detect dysfunction, monitor disease progression, and evaluate treatment effects in a range of retinal disorders [3]. ERGs are also increasingly applied to studies of ocular physiological processes such as emmetropization, the visually guided feedback mechanism that regulates postnatal eye growth to achieve and maintain emmetropia (good focus) [4–13]. Although substantial progress has been made in understanding the mechanistic principles of the emmetropization mechanism, the neural signals and pathways underlying retinal signaling of eye growth have yet to be fully elucidated [4, 5]. In this regard, ERGs can serve as an important tool for probing retinal neural pathways.

As a preclinical model, tree shrews, small semi-terrestrial, diurnal mammals closely related to primates, have gained significant popularity in neuroscience and vision science research [14–16]. This growing interest is primarily due to their phylogenetic proximity to primates, a well-developed brain with the highest brain-to-body mass ratio of any mammal including humans, and a visual system that resembles that of humans in many aspects [17, 18]. Tree shrews have excellent vision for a small mammal (visual acuity: 2–3 cycles/degree)[19], a cone-dominated retina, containing approximately 95% cones, and visual pathways and cortical structures that share key similarities with the primate visual system [17, 20–25]. These features have made tree shrews a commonly used model for studying refractive development, retinal information processing, and retinal diseases [18, 20, 26–28]. Their rapid developmental timeline, short breeding cycles [29], human-like fibrous sclera [30, 31], well-documented responses to visual manipulations such as defocus [32, 33], form deprivation [34], changes in light intensity [35, 36], variations in spectral composition [35, 37, 38], and experimentally induced human-like myopia [39], make them an ideal mammalian model for longitudinal studies of emmetropization and retinal physiology.

Despite their increasing use, electrophysiological assessments of retinal function in tree shrews have typically relied on large, stationary Ganzfeld systems [21, 28]. These instruments provide reliable recordings but are expensive, non-portable, and require dedicated laboratory facilities. The RETeval system (LKC Technologies, USA) is a compact and portable electroretinography device with a mini-Ganzfeld that offers practicality and operational simplicity for electroretinography. Although this system is widely used in human electrodiagnostics, its suitability and performance in tree shrews have not been characterized. The reliable use of the RETeval system in this species first requires an assessment of intra-session and inter-session repeatability, particularly for longitudinal studies conducted during the rapidly changing postnatal emmetropization period. These repeatability metrics are critical for distinguishing true physiological variability from random noise introduced by the instrument or recording conditions.

The purpose of this study was to evaluate the repeatability of ERG recordings obtained with the RETeval system in emmetropizing tree shrews. We quantified intra-session and inter-session repeatability across several commonly used ERG protocols, including photopic flash and flicker, S-cone, photopic negative response (PhNR), and On–Off ERGs. These findings provide the first systematic assessment of RETeval ERG repeatability in tree shrews and support the application of this device in investigations of retinal function and visually guided eye growth in this species.

## Methods

Twelve northern tree shrews (*Tupaia belangeri*) raised by their mothers at the University of Alabama at Birmingham (UAB) Tree Shrew Core were the subjects in this study. Tree shrews typically open their eyes at 3 weeks, which we designate as the first day of visual experience (DVE). By 24 DVE, they complete their initial rapid phase of emmetropization and achieve a stable refractive state of low hyperopia (approximately 1.4 D [33]). After 24 DVE, as the eyes grow during adolescence, the emmetropization mechanism continues to maintain emmetropia, exhibiting robust responses to visual cues. For example, if a negative power lens is placed over one eye at 24 DVE, moving the focal plane away from the retina, the emmetropization mechanism increases the axial length toward the shifted focal plane and fully compensates for the imposed lens to restore emmetropia (measured with the lens in place) by 35 DVE [40]. Therefore, we studied retinal function from 24 to 35 DVE.

All twelve animals came from different litters. They were raised under broadband light (100–300 lx) in a temperature-controlled colony maintained on a 14/10-h light/dark cycle starting at 6:30 AM. At 22 ± 1 DVE, a dental acrylic pedestal was installed onto the dorsal skull of the animal to facilitate handling and ocular alignment during autorefraction measurements in awake animals [41]. The animal care in this study adhered to the Association for Research in Vision and Ophthalmology (ARVO) guidelines for the use of animals in ophthalmic and visual research. All study protocols were approved by the UAB Institutional Animal Care and Use Committee.

### Retinal electrophysiology

Animals were anesthetized before recording the ERGs. The initial anesthesia was administered using 4% isoflurane gas for 3 min at a flow rate of 1000 ml/min, released inside the nest tube (used for transferring animals from the housing cage to the measurement room) containing the animal. The dormant animal was additionally administered an intramuscular injection of ketamine (100 mg/kg) and xylazine (7.5 mg/kg) in combination, based on body weight. The anesthetic effect of this combination drug lasts for approximately 45–60 min. After anesthesia, the animal was transferred to a temperature-regulated pad to maintain body temperature at 37 °C, which was continuously monitored using a rectal probe.

**Fig. 1:**
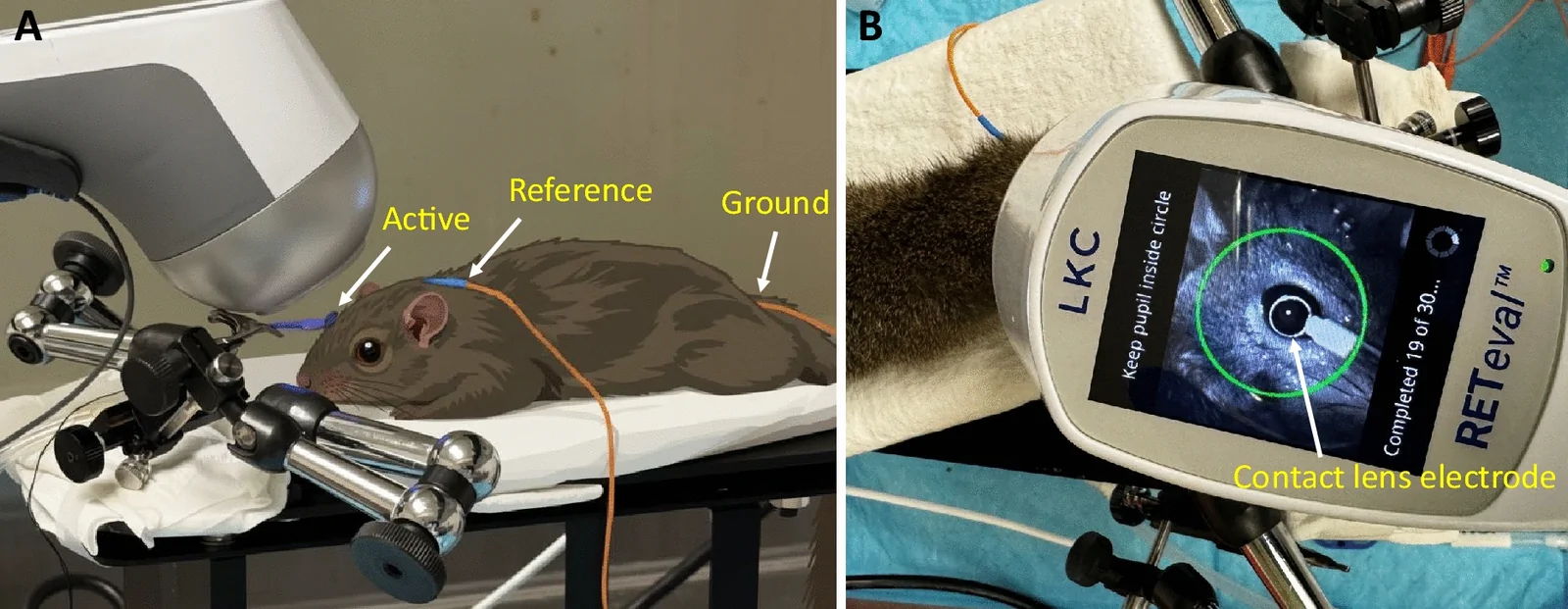
Electroretinogram testing using the RETeval system in tree shrews. (**A**) Location of ground, reference, and active electrodes. (**B**) Positioning of a contact lens (active electrode) on the cornea of an anesthetized tree shrew eye

Twenty-five minutes prior to the start of the ERG measurements, the pupil was dilated by instilling one drop of 2.5% phenylephrine (Paragon BioTeck, Inc., Portland, OR, USA). The animal was then placed on the two-channel rodent manipulator table, and the regions of attachment of electrodes were cleaned using 70% isopropyl alcohol prep pads. The ground and reference electrodes were 14 × 0.38 mm (length × diameter) stainless steel needles (Natus Neurology Incorporated, WI, USA, https://natus.com/neuro/natus-ultra-disposable-subdermal-needle-electrodes/). The ground electrode was inserted subcutaneously at the base of the animal’s tail. The reference electrode was inserted subcutaneously on the scalp behind the pedestal at the approximate intersection between the midline of the head and the midpoint of the ears (Fig. 1). The active electrode was an R250 rat contact lens electrode (LKC Technologies, MD, USA, https://store.lkc.com/rat-electrode/https://store.lkc.com/rat-electrode/) attached to a gold wire and placed in contact with the cornea, and coupled with 1% carboxymethylcellulose sodium (Refresh Liquigel, Allergan, AbbVie Inc., North Chicago, Illinois, USA). The recording eye was anesthetized with one drop of 0.5% proparacaine hydrochloride (Bausch + Lomb, Bausch & Lomb Incorporated, Tampa, FL, USA) before the contact lens electrode was placed.

The full-field photopic ERGs were recorded monocularly with the RETeval system (LKC Technologies, MD, USA) following the standards and extended protocols of the International Society of Clinical Electrophysiology of Vision [42–45]. The device was placed on the mounting arm with the mini-Ganzfeld in close proximity to the eye for full-field stimulation. Measurements were made at three different time points: 24, 30, and 35 DVE. We chose this juvenile period because animal studies probing the emmetropization mechanism have mostly been conducted during this period. Before starting the ERGs, the animals were light-adapted for 30 min under room illumination (100–300 lx). The protocols used were photopic flash, flicker, PhNR, S-cone, and On–Off ERGs. The flash and flicker protocols involved white flashes (3 cd.s/m^2^) against a white background (30 cd/m^2^) at frequencies of 2 and 28 Hz, respectively. The PhNR protocol used a red stimulus (621 nm peak, 1 cd.s/m^2^) against a blue background (470 nm, 10 cd/m^2^) flashed at a frequency of 3 Hz. In the On–Off protocol, the retina was stimulated with red flashes (621 nm, 560 cd.s/m^2^, 209.4 ms) against a green background (530 nm, 160 cd/m^2^) at a frequency of 2.4 Hz. In the S-cone protocol, a blue stimulus of 1 cd.s/m^2^ strength was flashed against a red background of 560 cd/m^2^ at a frequency of 4 Hz. Flash ERGs were quantified in terms of the amplitude and peak times of the a- and b-waves. Additionally, d-wave amplitude and peak times were analyzed for On–Off ERGs. The flicker ERGs were quantified in terms of the amplitude and peak time of the second peak. For the S-cone ERGs, the amplitude and peak times of the first positive peak (L/M cone response) and the second positive peak (S-cone response) were analyzed. The amplitude of the PhNRs was measured at 72 ms.

To investigate the intra-session repeatability, flash and flicker protocols were repeated twice at 24 DVE. The inter-session repeatability was assessed by comparing ERGs across 24, 30, and 35 DVEs. All measurements were performed by a single experienced examiner to avoid inter-examiner variability. After completion of the ERG measurements, animals were allowed to recover from the effects of anesthesia and were subsequently transferred back to their individual cages in the colony.

### Statistical analysis

The statistical analyses were performed using GraphPad Prism (v. 10.0.2, Boston, MA, USA) and SPSS Statistics (v. 28.0.1.1, IBM Corp., NY, USA). For S-cone ERGs, the second positive peak corresponding to S-cone function could be reliably obtained from six animals, so only these data were included in the analysis. The intra-session repeatability was assessed by comparing the ERG measurements between the two sessions obtained at 24 DVE. The inter-session repeatability was assessed by comparing measurements across different time points (24, 30, and 35 DVE). Repeatability metrics included the coefficient of variation (CoV), intra-class correlation coefficient (ICC), mean bias (%), and paired t-test (for inter-session repeatability) or one-way repeated measures analysis of variance (for inter-session repeatability). The CoV was calculated as the ratio of repeated measurement standard deviation to the mean, where a lower CoV indicated higher repeatability. The ICC is a measure of reliability index that measures both the correlation and agreement between repeated measurements. The ICC ranges between 0 and 1, where values between 0.75 and 0.9 represent good reliability and values > 0.9 indicate excellent reliability [46]. A negative ICC may be possible, which signifies zero reliability; in such a case, the value was reset to zero [47]. The ICC was calculated for absolute agreement using a two-way random measure. The 95% limits of agreement (LOA) between two sessions at 24 DVE (intra-session repeatability) and between 24 and 30 DVE sessions (inter-session repeatability) were calculated as the mean bias ± 1.96*SD. A tighter 95% LOA indicates better repeatability [48]. The mean bias percentage was calculated as the absolute difference between two repeated measurements divided by the mean of these measurements, multiplied by 100. This result is interpreted similarly to that of the CoV, where a lower value indicates greater repeatability. The effect of age on ERGs was evaluated using one-way repeated measures ANOVA, followed by post-hoc pairwise comparisons with Sidak correction for multiple testing. The data are presented as the means ± standard errors of the mean (SEMs) unless stated otherwise. A *p*-value of < 0.05 was considered statistically significant.

## Results

### Intra-session repeatability

**Fig. 2:**
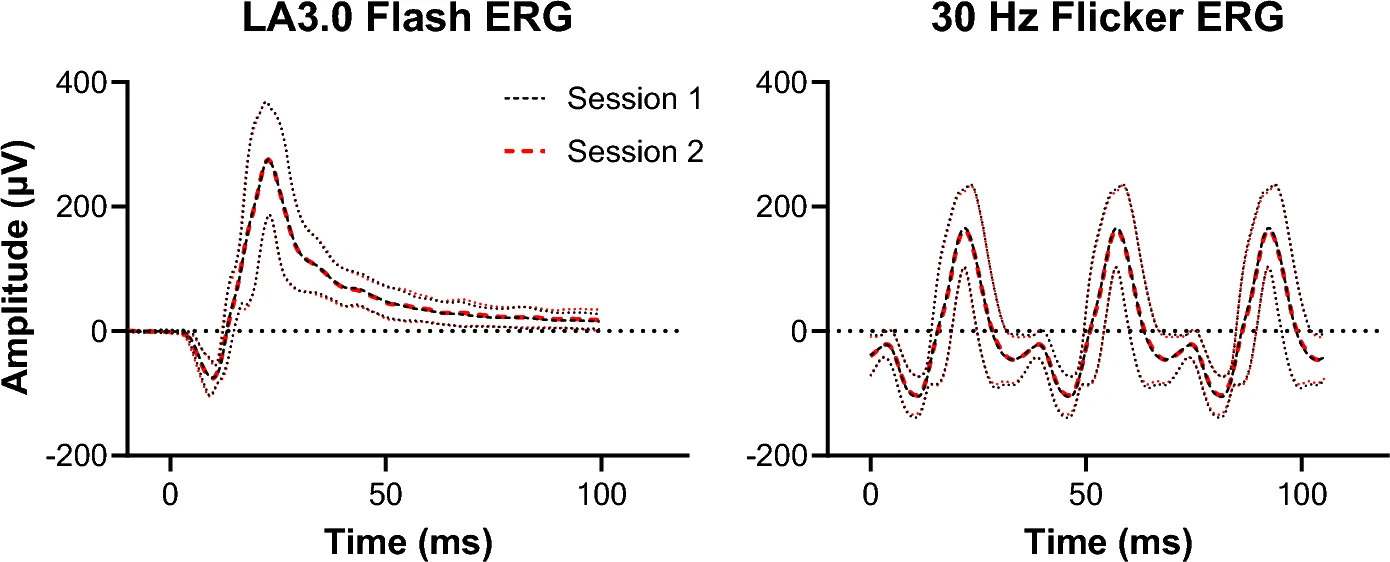
Flash and flicker electroretinograms obtained at the first (black dashed line) and second (red dashed line) 24 DVE sessions averaged across all animals. The dotted lines represent ± 1 standard deviation

Table 1 summarizes the CoV, ICC, mean bias (%), and paired t-test results for the amplitude and peak time measurements of the flash and flicker ERGs. All parameters had a CoV less than 5% and an ICC ≥ 0.98, indicating excellent intra-session repeatability. As illustrated in Figs. 2 and S2, the between-subject variability was also relatively low, suggesting good repeatability of ERGs across animals. Likewise, the mean bias was ≤ 6% across all parameters, and the paired t-tests were non-significant (*p* > 0.05) except for the flicker amplitude (*p* = 0.04). Figure 2 shows the average (mean ± SD) ERG waveform across all animals for the two sessions at 24 DVE. The ERG waveforms overlapped extensively, showing excellent intra-session repeatability.

**Table 1:** Intra-session repeatability of flash and flicker ERG peak times and amplitudes

		CoV (%)	ICC	Mean bias %	*P*-value^a^
Flash a-wave	Peak time	1.31	0.99**	1.85	0.89
	Amplitude	3.63	0.98**	5.14	0.31
Flash b-wave	Peak time	0.63	0.99**	0.88	0.14
	Amplitude	1.95	0.99**	2.76	0.57
Flicker waveform	Peak time	0.20	0.99**	0.28	0.37
	Amplitude	2.55	0.99**	3.61	0.04

*CoV* coefficient of variation, *ICC* intra-class correlation coefficient. ** represents *p* < 0.01. ^a^T-test

### Inter-session repeatability

**Fig. 3:**
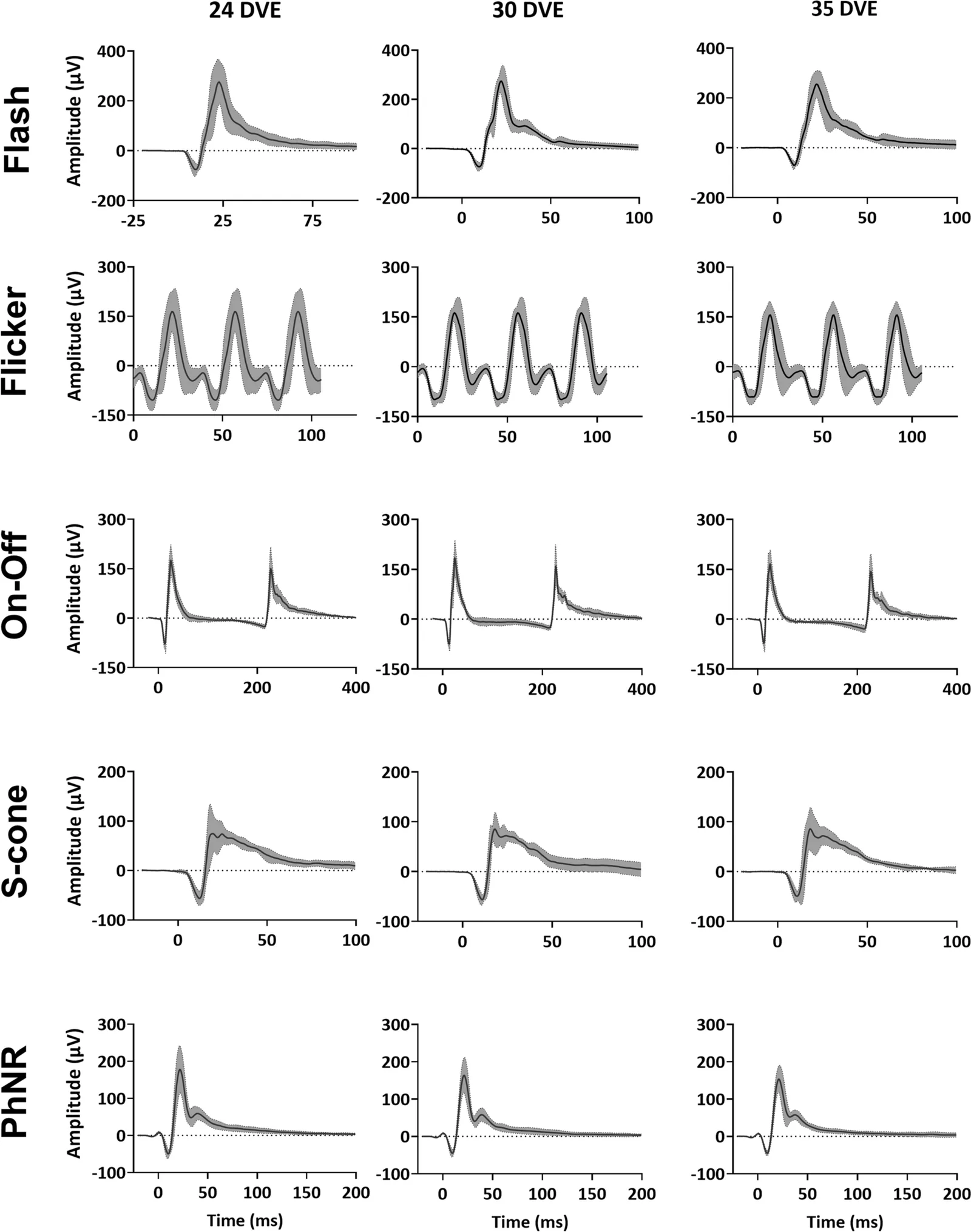
Full-field flash electroretinograms (ERGs) at 24, 30, and 35 days of visual experience (DVE) in tree shrews. Data are the average of all animals. The shaded region represents ± 1 standard deviation. PhNR: Photopic Negative Response

Table 2 summarizes the CoV, ICC, mean bias (%), one-way repeated-measures ANOVA, and Bland–Altman results for amplitudes and peak times across different ERG protocols. Inter-session CoV was lower for peak time responses (< 10%) than for amplitude responses (range: 13.36–29.35%) across all protocols. Similar results were observed for the mean bias (peak times: < 15%, and amplitudes: 18.89–41.51%). Unlike intra-session repeatability, only a few parameters had statistically significant ICC values (Table 2), and all but the S-cone amplitude (0.86) reached 0.75, indicating modest inter-session reliability of amplitudes and peak times. One-way repeated-measures ANOVA showed no difference in ERG peak times or amplitudes across the three DVEs. Likewise, Bland–Altman analyses showed smaller mean biases, with a lower 95% LOA range for peak time responses than for amplitude responses for all protocols. Figure 3 shows the average ERG waveforms recorded on 24, 30, and 35 DVE, and Fig. S1 shows the raw waveform for each animal. Qualitatively, the ERGs exhibited similar waveform patterns and morphology across the three time points.

**Table 2:** Inter-session repeatability of full-field flash ERGs in tree shrews

Protocol	CoV (%)	ICC (95% CI)	Mean bias %	*P*-value^a^	Mean bias ± 95% LOA
*Flash*					
a-wave peak time	7.48	0.52 (− 0.37,0.86)	10.58	0.65	− 0.23 ± 2.9
a-wave amplitude	13.36	0.66 (0.08,0.9)*	18.89	0.33	1.92 ± 46.6
b-wave peak time	5.87	0.54 (− 0.31,0.86)	8.30	0.62	− 0.47 ± 4.5
b-wave amplitude	13.57	0.50 (− 0.36,0.85)	19.20	0.42	− 19.78 ± 202
*Flicker*					
Peak time	2.84	0.61 (0.02,0.88)*	4.02	0.06	− 1.76 ± 4.4
Amplitude	14.70	0.55 (− 0.09,0.86)*	20.78	0.16	− 27.47 ± 153
*PhNR*					
Amplitude at 72 ms	29.35	0.68 (0.14,0.9)	41.51	0.19	− 3.57 ± 18.6
*S-cone*					
L/M cone peak time	6.78	0.51 (− 0.31,0.91)	9.58	0.08	− 1.63 ± 3.5
L/M cone amplitude	15.51	0.48 (− 2.3,0.93)	21.93	0.94	− 1.01 ± 87
S-cone peak time	8.69	0.43 (− 0.67,0.90)	12.29	0.21	− 2.83 ± 6.64
S-cone amplitude	22.02	0.86 (0.37,0.98)**	31.15	0.98	0.82 ± 21.86
*On–Off*					
a-wave peak time	7.20	0.59 (− 0.02,0.87)*	10.17	0.14	− 0.74 ± 3
a-wave amplitude	16.05	0.46 (− 0.39,0.83)	22.71	0.43	7.89 ± 46
b-wave peak time	7.34	0.40 (− 0.83,0.82)	10.39	0.70	− 0.7 ± 5.8
b-wave amplitude	14.30	0.44 (− 0.67,0.84)	20.22	0.83	0.44 ± 152
d-wave amplitude	19.00	0.74 (0.27,0.92)**	26.88	0.94	− 2.73 ± 126

*CoV* coefficient of variation, *ICC* intra-class correlation coefficient, *CI* confidence interval, *RM* repeated-measures, *LOA* limits of agreement

*and ** represent p < 0.05 and *p* < 0.01, respectively

^a^One-way repeated-measures ANOVA

### Effects of age

**Fig. 4:**
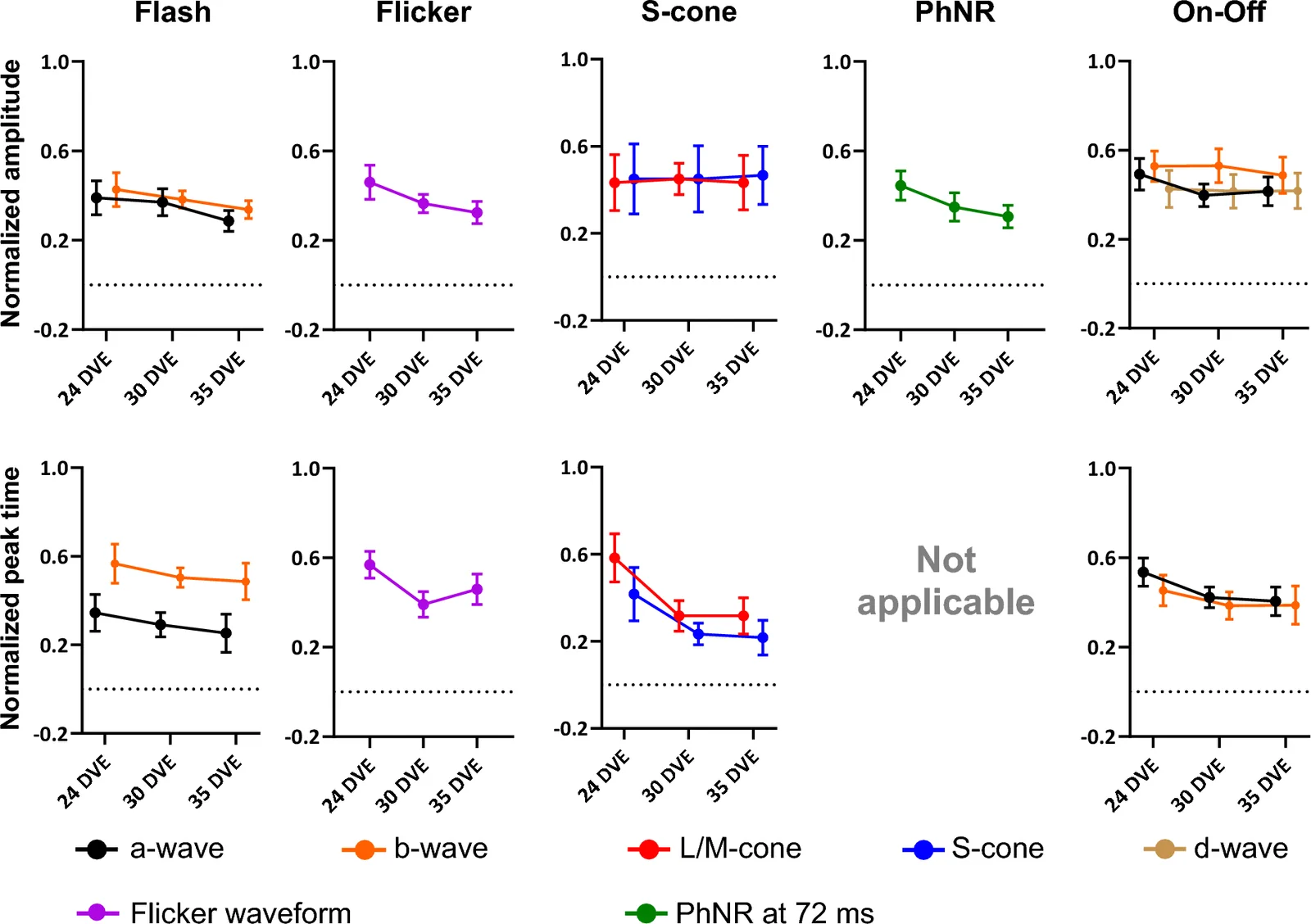
Effects of age on retinal function in tree shrews. The upper row represents amplitude responses, and the lower row represents peak time responses. Since PhNR was measured at 72 ms, peak time comparisons are not applicable. For each response component (e.g., a-wave), data are normalized between 0 and 1, and are shown as the mean ± 1 standard error of the mean

Figure 4 shows the average (mean ± SD) ERG response amplitudes and peak times for different protocols at 24, 30, and 35 DVE. One-way repeated measures ANOVA revealed no significant differences in flash ERG a-wave amplitude (*p* = 0.33) and peak time (*p* = 0.65), b-wave amplitude (*p* = 0.42) and peak time (*p* = 0.62), flicker ERG amplitude (*p* = 0.16) and peak time (*p* = 0.06), L/M-cone amplitude (*p* = 0.95) and peak time (*p* = 0.08), S-cone amplitude (*p* = 0.98) and peak time (*p* = 0.21), PhNR amplitude (*p* = 0.16), and on–off a-wave amplitude (*p* = 0.43) and peak time (*p* = 0.14), b-wave amplitude (*p* = 0.83) and peak time (0.70), and d-wave amplitude (*p* = 0.94). These results suggest that the RETeval values of photopic ERGs in tree shrews were relatively stable from 24 to 35 DVE.

### Comparison of tree shrew and human ERGs

**Fig. 5:**
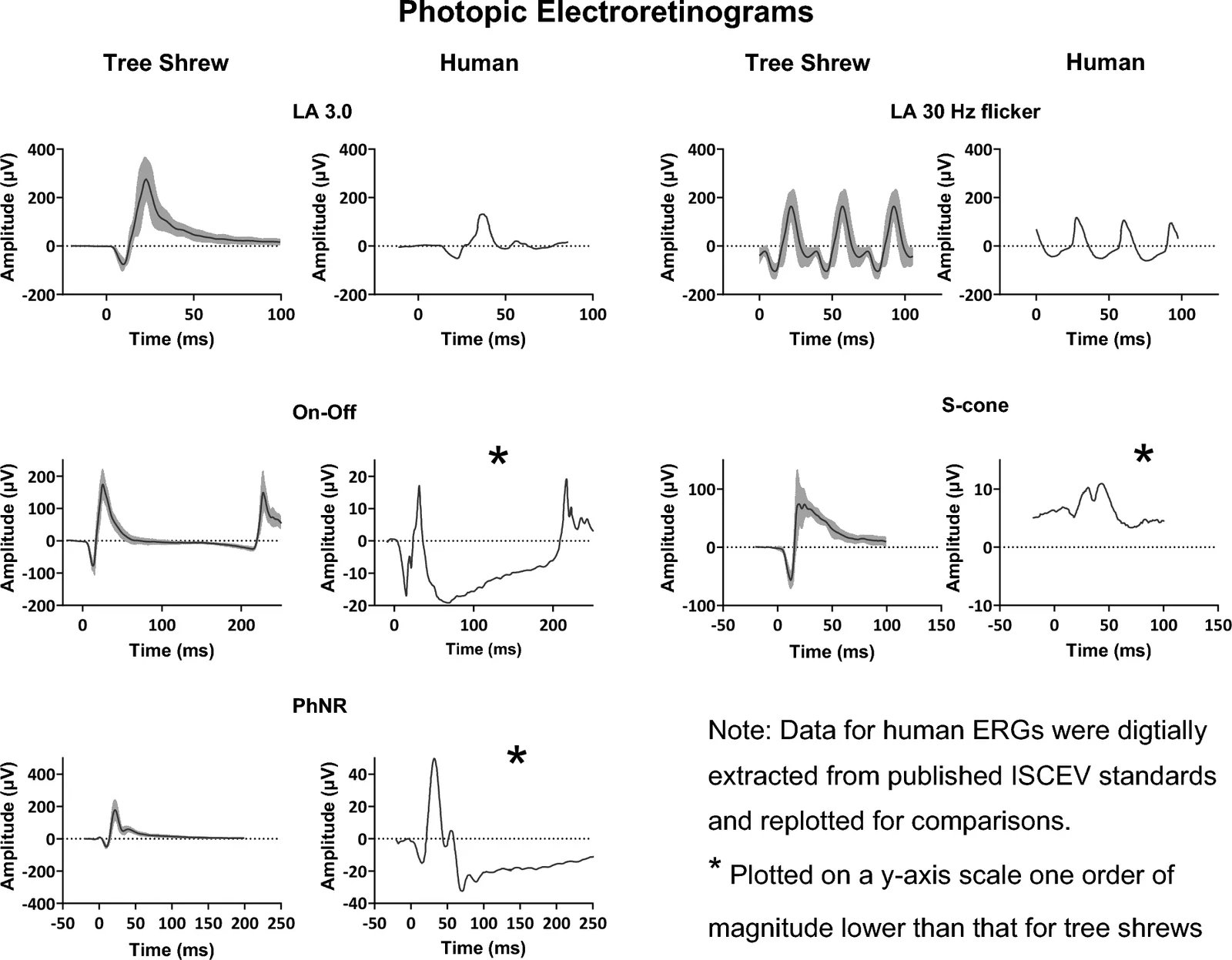
Comparison of tree shrew and human full-field photopic electroretinograms (ERGs). Tree shrew ERGs were obtained with the RETeval device using a contact lens electrode after light adaption for 10 min. Data for tree shrews are shown as the mean ± standard deviation (shaded grey region). Human ERGs for different protocols were replotted using data extracted from the corresponding published ISCEV standards [42–45]

We further evaluated the degree of congruency in the ERG waveforms between tree shrews and humans. Human ERG data for the different protocols were extracted from the corresponding ISCEV standards using WebPlotDigitizer (v5, https://automeris.io/) and replotted on the same scale, or on a scale one order of magnitude lower, to allow comparison with tree shrew ERGs. As illustrated in Fig. 5, the waveform morphology of the photopic ERGs in tree shrews closely resembled that of the photopic ERGs in humans. However, noticeable differences were evident in both response magnitude and timing, despite matched stimulus and recording parameters. Specifically, the amplitudes of human photopic ERGs were considerably smaller than those of tree shrews, and in some cases by approximately an order of magnitude (e.g., S-cone, PhNR, and on–off ERGs). Additionally, the peak times of the a- and b-waves of the photopic ERGs were consistently shorter in tree shrews than in humans. We also measured scotopic ERGs in one representative animal following the ISCEV guidelines for scotopic full-field ERGs [45] and compared these responses with the human ERGs published in the ISCEV standard [45]. Compared to human ERGs, the amplitudes of tree shrew scotopic ERGs were smaller, and the peak times were shorter (Fig. S2), although this result remains preliminary and warrants further investigation.

## Discussion

This study represents the first systematic evaluation of retinal function in tree shrews using the mini-Ganzfeld RETeval system. All photopic ERG protocols, including flash, flicker, PhNR, On–Off, and S-cone recordings, produced clear and well-defined waveforms with robust components. The results serve as a useful reference for other groups using tree shrews as a mammalian model for visual neuroscience research and provide a strong foundation for investigations of neural mechanisms underlying retinal health, physiology, and diseases.

Intra-session analyses confirmed strong repeatability for flash and flicker protocols, as indicated by low coefficients of variation, minimal mean bias, high intraclass correlation coefficients, and non-significant paired comparisons. Although a small difference in flicker amplitude was detected between repeated measures, other responses were largely stable and repeatable. Inter-session repeatability was relatively modest with greater variability. Peak times were more consistent across days than amplitudes, consistent with the typically greater between-session variability in amplitudes [49]. The poorest repeatability was observed in the S-cone and PhNR protocols, likely due to the smaller number of animals with a clearly identifiable S-cone peak (n = 6) and an arbitrary single time-point (72 ms) analysis for PhNR amplitude for consistency. Additionally, the PhNR trough was less clearly defined in most cases, presumably due to the effects of prior gas anesthesia (during animal handling before starting the ERG measurements) on retinal ganglion cell activity [50].

A key strength of this manuscript is the use of multiple statistical metrics, including CoV, ICC, mean bias, and repeated-measures ANOVA, to evaluate the intra- and inter-session repeatability of ERGs in tree shrews [51]. Tree shrews are diurnal and phylogenetically closer to humans than rodents, with fundamental differences in retinal organization [52, 53]. Therefore, direct comparisons of absolute ERG amplitudes and peak times are less appropriate, but comparisons of repeatability metrics are still informative. Using contact lens electrodes, Bayer and colleagues reported the inter-session repeatability of photopic flash ERG responses in seven adult mice (6 months of age) and rats (5 months of age) over five consecutive days [54]. Under photopic conditions, the mean CoV for b-wave amplitude was 5.6% (range: 3.1–8.1%) in mice and 10.6% (range: 5.8–15.4%) in rats. Our study showed a slightly greater mean CoV (13.6%, range: 0.28–27.9%) in tree shrews. Consistent with these findings, the ICCs for flash ERG b-wave amplitude were lower in tree shrews (peak time: 0.54; amplitude: 0.50) than in rats (peak time: 0.84; amplitude: 0.63) but comparable to those in mice (peak time: 0.58; amplitude: 0.60) [54].

The discrepancy in repeatability metrics between tree shrew and rodent ERGs could be partly attributed to age differences. Baylor et al. tested mice at 5–6 months, but the tree shrews in the present study were slightly younger (~ 6 weeks postnatal, 24 DVE). Age is known to be a critical biological variable that influences ERG activity in both humans [55, 56] and mice [55, 57]. Park et al.[58] recorded longitudinal ERGs in ten male mice from 5 to 70 weeks of age and showed a decrease in amplitudes and an increase in peak times with age. Interestingly, the amplitude changes were non-linear, with an initial decrease from 5 to 30 weeks, a slight increase between 30 and 50 weeks, and a subsequent decrease after 50 weeks. In contrast, our results showed no effect of age on the amplitudes and peak times of photopic ERG components during the early juvenile period (from 24 to 35 DVE) in tree shrews (Fig. 4). Although the lack of age-related effects on ERGs could be due to the narrow age range of animals, retinal function may also remain relatively stable during early postnatal development in tree shrews, despite rapid concurrent structural and physiological changes [59, 60]. Given the minimal influence of age, methodological and physiological factors likely contributed to the relatively greater inter-session variability in this study. These factors may include the variable degree of coupling between the cornea and electrode, electrode placement and impendence, or systematic physiological noise across different measurement sessions [49]. Despite some variability, the waveform morphology remained consistent across sessions (see Fig. 3), indicating stable retinal functional integrity in tree shrews during the juvenile period.

The photopic ERGs obtained from tree shrews were qualitatively similar in waveform morphology to those of humans. However, there were measurable differences in the magnitude and timing of the responses, which interacted with the state of light adaptation. Photopic ERG amplitudes in tree shrews were substantially greater than those in humans, whereas scotopic ERG amplitudes were considerably lower. For some protocols, such as the S-cone, On–Off, and PhNR, the difference was approximately an order of magnitude greater. In addition, the photopic ERGs from tree shrews exhibited shorter peak times compared to those in humans. The amplitude differences could be attributed, at least in part, to the difference in eye size between the two species. The axial length of the tree shrew eye is approximately 70% shorter than that of the human eye (approximately 7.5 mm vs 24 mm) [61]. In addition to the closer proximity of the recording electrode to the retina, the same stimulus strength in a relatively smaller tree shrew eye may produce greater retinal illuminance than in a human eye, causing greater amplitudes of photopic ERGs. A shorter eye could also allow a faster attainment of the neural activation threshold due to higher effective retinal illuminance, potentially resulting in shorter ERG peak times. Moreover, as fast-moving creatures, tree shrews are known to exhibit faster perceptual responses than humans [62].

Alternatively, the discrepancy in ERG amplitudes and peak times between tree shrews and humans could be explained by differences in the types and distribution of photoreceptors. Tree shrews are dichromatic with two cone types: short-wavelength-sensitive (SWS) cones (peak sensitivity, λ_max_ = 428 nm) and long-wavelength-sensitive (LWS) cones (λ_max_ = 555 nm) [63]. While most humans are trichromats with additional middle-wavelength-sensitive cones (λ_max_ = 534 nm), they have similar peak sensitivities of SWS (λ_max_ = 420 nm) and LWS (λ_max_ = 563 nm) cones [64]. Tree shrews possess a cone-dominated retina (~ 95% cones), with densities ranging from 12,000/mm^2^ in the superior far periphery to 36,000/mm^2^ in the inferonasal periphery, with no specific local maxima at the central retina [24]. In contrast, humans have cone dominance only in the foveal region (local maxima), which comprises ~ 5% of the total photoreceptors, and the cone density varies from as high as ~ 199,000/mm^2^ (on average) in the fovea centralis to 10,000/mm^2^ at 20 degrees eccentricity [65–67]. The human retina is rod-dominated outside the fovea, likely explaining the stronger scotopic responses. Rod density in tree shrews ranges from 200 rods/mm^2^ in the superior retina to 3000–3500/mm^2^ in the inferior far periphery [24], whereas in humans, the fovea centralis is rod-free, and the highest rod density is found in the superior region of an elliptical ring at the eccentricity of an optic disc (176,000/mm^2^) [67]. Despite these anatomical differences, the close resemblance of the morphology of the tree shrew ERG waveform to that of humans underscores the translational relevance of using the tree shrew as an animal model for studies on retinal physiological mechanisms as well as retinal health and disease.

In conclusion, the portable RETeval system provides reliable measurements of ERGs in tree shrews, with excellent intra-session repeatability and modest inter-session repeatability. Despite inter-species differences in response magnitude and timing, the waveform morphology of photopic ERGs in tree shrews closely approximates that of human photopic ERGs. These findings support the use of portable ERG systems in tree shrews and lay a foundation for future applications, including studies of retinal function, disease models, and emmetropization mechanisms in this species.

## Supplementary Information

Below is the link to the electronic supplementary material.

Supplementary file1 (DOCX 1015 KB)

## Data Availability

The datasets generated during the study are available from the corresponding author upon reasonable request.

## Declarations

### Statement on the welfare of animals

All applicable international, national, and/or institutional guidelines for the care and use of animals were followed. All experimental procedures using animal models were described and approved by the Institutional Animal Care and Use Committee at the University of Alabama at Birmingham (UAB) (Protocol #22448).
