# Supplementary material for: Measuring electroretinograms in tree shrews with a portable electrophysiology device
Source: Doc Ophthalmol. 2026 Mar 30;153(2):165–77. doi: 10.1007/s10633-026-10095-w (PMC13619756; doi:10.1007/s10633-026-10095-w)
Supplement: Supplementary file 1 [file 10633_2026_10095_MOESM1_ESM.docx]

**Supplementary Material**

**Measuring Electroretinograms in Tree Shrews with the RETeval System**

Rohit Dhakal* (ORCID ID: 0000-0002-7722-7065)

Nilda Sanchez-Castellanos* (ORCID ID: 0009-0000-0486-7179)

Timothy J Gawne (ORCID ID: 0000-0002-5634-4393)

Safal Khanal^#^ (ORCID ID: 0000-0002-3755-8046)

Department of Optometry and Vision Science, University of Alabama at Birmingham (UAB), Birmingham, AL, United States

*Co-first authors

^#^ Corresponding author

Dr. Safal Khanal

Department of Optometry and Vision Science

University of Alabama at Birmingham (UAB)

HPB 526, 1716 University Blvd.

Birmingham, AL 35233, United States

Email: [skhanal@uab.edu](mailto:skhanal@uab.edu)

Figure S1. Full field photopic electroretinograms (ERGs) at 24, 30, and 35 DVE for all animals. Each trace represents data from an individual animal. Data for flash, flicker, On-Off, and Photopic Negative Response (PhNR) are from 12 animals. Data for S-cone ERG are from 6 animals.

Figure S2. Comparison of tree shrew and human full-field scotopic electroretinograms (ERGs). Tree shrew ERGs were obtained with the RETeval device using a contact lens electrode after dark adaption for 20 minutes. Human ERGs were replotted using data extracted from the published ISCEV standard for dark-adapted full-field ERGs (Robson et al. 2022).
